# Using a Natural Triterpenoid to Unlock the Antitumor Effects of Autophagy in B-Cell Lymphoma

**DOI:** 10.3390/biomedicines13020445

**Published:** 2025-02-12

**Authors:** Bently P. Doonan, Faisal F. Y. Radwan, Naren L. Banik, Azizul Haque

**Affiliations:** 1 Department of Medicine, University of Florida, Gainesville, FL 32610, USA; bently.doonan@medicine.ufl.edu; 2 Department of Pharmacology and Immunology, Medical University of South Carolina, Charleston, SC 29425, USA; radwan.faisal@gmail.com (F.F.Y.R.); baniknl@musc.edu (N.L.B.); 3 Cancer Biology and Immunology, Hollings Cancer Center, Medical University of South Carolina, Charleston, SC 29425, USA; 4 Department of Neurosurgery, Medical University of South Carolina, Charleston, SC 29425, USA; 5 Oncology, Spinal Cord Injuries and Disorders, Ralph H. Johnson Veterans Administration Medical Center, Charleston, SC 29401, USA

**Keywords:** B-cell lymphoma, triterpenoid, autophagy, mitochondrial integrity, apoptosis, chemotherapy, immunotherapy

## Abstract

**Background and Objective:** Diffuse large B-cell lymphoma (DLBCL), a subtype of non-Hodgkin’s lymphoma, is the most common lymphoid malignancy in the Western world. Treatment of DLBCL has been greatly improved in recent years with the addition of the monoclonal antibody Rituximab to the gold standard CHOP (cyclophosphamide, doxorubicin hydrochloride, vincristine sulfate, and prednisone) chemotherapy regimen, but these treatments are often ineffective in patients with highly aggressive disease or patients of advanced age. While CAR-T cells have further advanced the treatment landscape of DLBCL, these often come at significant costs such as toxicity and financial costs for patients. Thus, research has recently focused on natural products that can selectively target malignant lymphomas while displaying a reduced host toxicity profile. **Methods:** In vitro cellular and biochemical approaches were used to analyze the effects of a natural extract from the *Ganoderma lucidum* mushroom (GA-DM) on autophagy and apoptosis in human and mouse B-cell lymphoma lines. In addition, in vivo approaches were applied to determine the effect of GA-DM on tumor growth and metastasis in a mouse model of B-cell lymphoma. **Results:** Here, we report, for the first time, that GA-DM induces apoptosis in the human B-cell lymphoma cell lines DB and Toledo, and orchestrates autophagy and apoptosis in the murine B-cell lymphoma cell line A20. While GA-DM differentially induced autophagy and apoptosis in mouse and human B-cell lymphomas, blocking apoptosis by the caspase inhibitor Z-VAD-FM reduced anti-proliferative activity in human B-cell lymphoma cells (DB: 71.6 ± 6.2% vs. 56.7 ± 2.4%; Toledo: 53.1 ± 10.6% vs. 14.6 ± 9.3%) in vitro. Antitumor efficacy of GA-DM was also investigated in vivo in a murine B-cell lymphoma model using the A20 cell line, where GA-DM treatment reduced both the number of tumor metastases (control: 5.5 ± 3.2 vs. GA-DM: 1.6 ± 0.87) and the overall tumor burden (control: 3.2 g ± 1.9 vs. GA-DM: 1.70 g ± 0.2) in diseased mice. **Conclusions:** These findings support the potential use of GA-DM as a novel chemotherapeutic in the treatment of DLBCL and could improve the treatment of higher-risk patients with advanced disease who cannot tolerate current chemotherapy treatments.

## 1. Introduction

B-cell lymphomas are clonal tumors of mature and immature B-cells that constitute the majority of non-Hodgkin lymphomas (NHLs) [1,2,3]. In B-cell lymphoma, a monoclonal population of dysfunctional lymphocytes proliferates and grows out of control, crowding out normal cells and causing the lymph nodes to enlarge. In the Western world, diffuse large B-cell lymphoma (DLBCL) is the most common form of NHL and represents 85% of all lymphomas in the United States [1,4]. Common presentations of this disease include high peripheral white blood cell counts with lymphocyte predominance, painless enlargement of one or more lymph node areas, and so-called B symptoms of fever, night sweats, and weight loss [5,6,7]. There are approximately 7 new cases of DLBCL for every 100,000 people per year in the United States, with the population effect skewed toward older or elderly individuals [8,9]. Like other NHLs, DLBCL tends to affect white males more frequently than any other demographic group [9]. The clinical gold standard of treatment for DLBCL patients is R-CHOP and a combination chemo-immunotherapeutic approach using the anti-CD20 monoclonal antibody Rituximab in combination with chemotherapeutics [6,10]. This treatment plan has greatly improved patient outcomes over the last decade, and roughly 40% of patients are cured by this as the initial treatment [11]. Unfortunately, this means that 60% of patients experience disease recurrence or refractory disease unaffected by treatment [11]. The shortcomings of R-CHOP and the issue of treating an elderly patient population provide ongoing problems for the successful treatment of DLBCL. Recent advances in bi-specific antibody therapy, antibody–drug conjugates, immune-modifying agents, second-generation CD20-inhibiting monoclonal antibodies, and CAR-T cells have further advanced the treatment landscape of DLBCL, yet these often come at significant costs, both in toxicity and financial costs, that prevent all patients, particularly the elderly, from accessing them [12,13].

Autophagy and apoptosis play crucial roles in the killing of malignant cells and regulating immune recognition in the host [14,15,16]. Defects in these processes may cause or contribute to infectious, autoimmune, or neoplastic diseases. While tumors use multiple mechanisms to escape from apoptosis and immune detection [17,18], active involvement of autophagy and apoptosis and their orchestrated crosstalk in this scenario could be important. However, some tumors respond differentially to autophagy and apoptosis induced by chemotherapeutics. Recently, much effort has been placed on the discovery of natural plant products/extracts that display a more favorable toxicity profile than traditional chemotherapeutics [19,20,21,22]. One attractive source of antitumor products is the *Ganoderma lucidum* mushroom, which has been used for centuries in East Asia as an herbal treatment for inflammatory and malignant diseases, viral infections, and many other disease conditions [23,24,25]. Our laboratory has recently shown that one extract of the *G. lucidum* mushroom, Ganoderic Acid DM (GA-DM), has the ability to kill melanoma cells while stimulating the immune system both in vitro and in vivo [16]. However, the effect of GA-DM on DLBCL, an aggressive NHL, remains unknown.

While chemotherapeutic drugs induce cellular toxicity [26,27], in many cases, they are used as a general mainstay of cancer treatment, destroying cancer cells by inducing apoptosis [28], which effectively reduces the size of the tumor and prevents further tumor growth. Autophagy is also crucial for the recycling of cellular components to generate nutrients and to enable survival in times of cellular stress [29,30]. However, studies suggest that excessive autophagy may lead to cell death, with the hallmarks typical of apoptotic events [31,32]. Crosstalk between autophagy and apoptosis could regulate cancer cell death and survival and trigger immune activation. One such agent could be triterpenoid, which has potent antitumor activity and which may induce immune components in malignant B-cells. The majority of B-cell lymphomas express MHC class II molecules, which can present tumor-derived peptides from cells undergoing apoptosis and enhance antitumor immune responses. Understanding the multifactorial function of this natural plant product could help in the design of new anti-cancer drugs, which may sensitize cancer cells and activate immune responses, eliminating the malignant growth of B-cell lymphomas.

A significant gap in lymphoma therapy currently exists for patients who develop resistance to standard treatments, particularly when dealing with relapsing lymphomas, where limited options remain after initial therapies fail. Thus, there is an unmet need for a better understanding of resistance mechanisms to develop more targeted and effective treatment strategies, especially for specific patient populations where the long-term side effects of treatment are a major concern. Both autophagy and apoptosis can have a role in the suppression of tumor growth because excessive autophagy promotes the death of tumor cells, while apoptosis prevents their survival. A complex array of biomarkers is involved in maintaining the coordination between survival and death pathways, where the crosstalk between autophagy and apoptosis may dictate the fate of malignant cells [30,33]. Although autophagy and apoptosis are thought to be two different forms of programmed cell death in antitumor therapy, they have a complex relationship, with autophagy sometimes acting as a protective mechanism for cancer cells [33,34,35]. This could hinder the apoptosis induced by a treatment. In other situation, inducing autophagy by a natural product can enhance the efficacy of anti-cancer drugs by promoting cell death, depending on the cancer type and treatment conditions. Thus, understanding this interplay is crucial for developing effective cancer therapies.

In this study, mouse and human lymphoma cells were tested using the natural triterpenoid GA-DM, which showed differential sensitivity in these tumor lines and in cell-death pathways. Specifically, this study found that GA-DM is cytotoxic to DLBCL cell lines in vitro and investigated the mechanisms of GA-DM-induced apoptotic and autophagic cell death in vitro and in vivo. The data obtained indicate that GA-DM treatment induces autophagic and apoptotic events in mouse and human DLBCL cell lines, respectively, through the induction of Beclin-1 and caspase 3 proteins. Mechanistic studies suggest that major autophagic and minor apoptotic events might be possible in a mouse B-cell lymphoma, whereas major apoptotic events could occur in human B-cell lymphoma lines. In vivo studies in an A20 BALB/c mouse model of B-cell lymphoma suggest that GA-DM treatment reduces the overall tumor burden and limits the number of liver metastases. Taken together, our current study suggests that GA-DM has great potential as a natural product capable of inducing autophagy and tumor destruction and for inhibiting the formation of tumor metastases in vivo.

## 2. Materials and Methods

### 2.1. Cell Lines

The mouse B-cell lymphoma line A20 was a gift from Dr. Yang Yiping (Duke University Medical Center, Durham, NC, USA). The human B-cell lymphoma lines DB and Toledo used in each experiment were obtained from the American Type Culture Collection (ATCC, Manassas, VA, USA) and cultured in complete RPMI (Invitrogen, Grand Island, NY, USA) plus 10%FBS (HyClone, Logan, UT, USA), penicillin/streptomycin (Mediatech Inc., Manassas, VA, USA) and 1% glutamine (Mediatech) in 5% CO_2_ at 37 °C [36,37].

### 2.2. Triterpenoids and Cellular Cytotoxicity Assays

The triterpenoid Ganoderic Acid DM (GA-DM), originally isolated from the *Ganoderma lucidum* mushroom, was purchased from Planta Analytica, LLC (Danbury, CT, USA) (Cat# G-032). The purity of GA-DM was determined by the vendor as 99.9% using LC/MS analysis. GA-DM was dissolved in DMSO (Sigma-Aldrich, St. Louis, MO, USA) to make a 10 mM stock solution for use in the cytotoxicity assay. For all GA-DM treatments, the final DMSO concentration was ≤1%. Cell lines were seeded at 1 × 10^5^ cells/well in 100 µL of the appropriate culture medium in a flat-bottom 96-well plate. A wide range of GA-DM concentrations have been tested in a number of cancer cell lines in our laboratory, which indicate that 10–40 µM GA-DM concentrations induce cell death (unpublished data). Thus, GA-DM was added to the appropriate wells at final concentrations of 0, 10, 20, 30, and 40 µM for 24 h at 37 °C. Following 24 h of GA-DM treatment, anti-proliferative activity was measured using the CellTiter 96 Aqueous One Solution Cell Proliferation Assay (MTS; Promega, Madison, WI, USA). Data are representative of at least three separate experiments and are expressed as the anti-proliferative activity from the measurement of cell viability (% of control) ± S.D. of triplicate wells.

### 2.3. Caspase Activation and Inhibition Assays

Lymphoma cell lines were treated with different concentrations (0, 10, and 20 μM) of GA-DM, and caspase activities were tested according to the manufacturer’s protocol (Promega Corporation, Madison, WI, USA; Cat-G8090). Briefly, 5 × 10^4^ cells were plated in a total volume 100 μL in a 96-well plate and treated with 0 (vehicle control), 10, or 20 µM of GA-DM for 24 h at 37 °C. The plate was then equilibrated for 30 min at room temperature, and 100 μL of Caspase-Glo^®^ 3/7 reagents (Promega Corporation, Madison, WI, USA) was added to each well. Luminescence was recorded 30 min after adding the reagents using a FLUOstar Optima microplate reader (BMG, Durham, NC, USA) as previously described [16].

Cells were also treated with 20 µM of GA-DM for 24 h in the presence or absence of a pan-caspase inhibitor (Z-VAD-FMK, 50 µM) (R&D systems #FMK001, Minneapolis, MN, USA). Following treatment, cell viability was measured using the CellTiter 96 AQueous One Solution Cell Proliferation Assay (MTS) [16,37]. Experiments were repeated at least three times, and the data were expressed as the percent cytotoxicity ± S.D. of triplicate wells.

### 2.4. Western Blot Analysis

Cell lines were cultured for 24 h in the presence of 0, 10, and 20 µM of GA-DM. Following treatment, cells were washed, and cell lysates were obtained using a standard lysis buffer. Equal protein concentrations from designated cell lysates (50 µg) were separated on a 4–12% Bis/Tris 12-well NuPage gel [16,38,39]. Proteins were transferred onto a nitrocellulose membrane (Pierce, Rockford, IL, USA) and probed with antibodies to detect the expression of caspase 3 and Beclin-1 (Santa Cruz Biotechnology, Louis, MO, USA). A monoclonal antibody against β-actin (Santa Cruz) was used as a protein loading control. Relative protein expression was assessed using ImageJ software, 1.54f, NIH (National Institutes of Health, Bethesda, MD, USA) and expressed as the relative density for each sample [16,36,37].

### 2.5. TMRE Staining

Cells treated with 0, 10 or 20 µM of GA-DM were stained with TMRE (tetramethylrhodamine ethyl ester, 200 nM) (Abcam, Boston, MA, USA) and analyzed by flow cytometry according to the manufacturer’s protocol to determine mitochondrial integrity [16].

### 2.6. In Vivo Tumor Induction and Evaluation

Tumors were induced by the intravenous injection of A20 cells (2 × 10^5^ cells; >90% viability by Trypan blue exclusion) into the tail veins of BALB/c mice. Treatment was initiated by intraperitoneal (i.p.) injection of GA-DM (50 mg/kg) or control vehicle (DMSO, Sigma) at day 10 and day 21 after A20 tumor implantation. The cell numbers for inducing tumors and the GA-DM dose have been optimized in our laboratory using several cell lines in vitro and mouse models in vivo (unpublished data). Following treatment, mice were kept under observation for signs of disease, weight loss, or erratic behavior, at which point they were requisitely euthanized. Tumor-bearing mice were sacrificed if they showed severe signs of any moving disability that hampered them from obtaining food and water. All other mice were euthanized starting at day 35 and analyzed for disease impact. Mouse livers were removed and weighed, and the number of liver metastases was recorded. Treatment or control groups consisted of 8 mice (*n* = 8), with 4 mice used as a naïve untreated group for a comparison with healthy liver tissues. All work with mice was approved by the Medical University of South Carolina Animal Protocols (IACUC-2018-00279, approved on 18 March 2021) Review Board and was performed in accordance with the National Institutes of Health *Guide for Care and Use of Laboratory Animals* [40].

### 2.7. Statistical Analysis

Data from each experimental group were subjected to statistical analysis. Statistical analyses were performed using Microsoft Excel and GraphPad Prism (version 6.0) software as described [41]. The immunoreactive bands obtained from Western blotting and the immunoreactive pixels of the immunofluorescence data were analyzed with ImageJ software, 1.54f, NIH (US National Institutes of Health, Bethesda, MD, USA). ANOVA with post hoc tests (Tukey’s honest significant difference test), repeated-measures ANOVA with post hoc tests or Student’s *t*-tests were also used as appropriate [16,38,39]. Two-sided tests were used in all cases, and *p* values < 0.05 were considered statistically significant. The average deviation for sample groups was calculated, and error bars are indicated.

## 3. Results

### 3.1. Anti-Proliferative Activity by the Natural Product GA-DM

**Figure 1:**
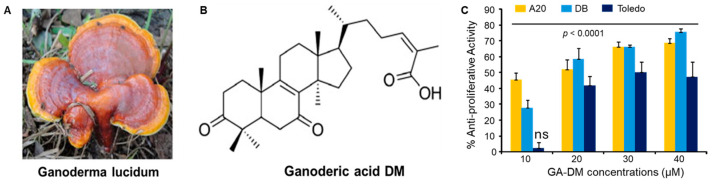
Anti-proliferative activity of Ganoderic Acid DM (GA-DM) from *Ganoderma lucidum*. (**A**) Photomicrograph of *Ganoderma lucidum* [42]. (**B**) GA-DM structure. (**C**) The mouse B-cell lymphoma line A20 and the human B-cell lymphoma lines DB and Toledo were incubated with various concentrations of GA-DM for 24 h. Anti-proliferative activity was measured by the MTS assay as described in the methods. Data are representative of three separate experiments. No significant difference in Toledo was observed at the 10 μM concentration compared to the vehicle control. All other treatment groups show statistically significant differences compared with the vehicle-treated controls. ns = not significant.

The effect of Ganoderic Acid-DM (GA-DM), a natural extract of the medicinal mushroom *Ganoderma lucidum* (Figure 1), was tested on B-cell lymphoma growth. The chemical structure of GA-DM is shown in Figure 1B. GA-DM has previously been tested in melanoma cells using a known apoptosis inducer, staurosporine, as a positive control [16]. In this study, the mouse B-cell lymphoma cell line A20 and the human B-cell lymphoma lines DB and Toledo were treated with serial doses of GA-DM (0–40 µM), and the anti-proliferative activity of GA-DM was tested by measuring the cell viability using the MTS assay [16]. Control vehicle-treated cells were used to calculate the percentage of anti-proliferative activity. The results show that GA-DM treatment reduced the viability of both human and mouse B-cell lymphomas in a dose-dependent manner. Treatment of cells with 10–20 µM GA-DM significantly reduced cell viability (Figure 1C), although the degree of sensitivity to GA-DM was different for each cell line. These data suggest that the natural extract GA-DM attenuates lymphoma cell growth in vitro.

### 3.2. GA-DM Differentially Activates Caspase Processing in Human and Mouse B-Cell Lymphomas

**Figure 2:**
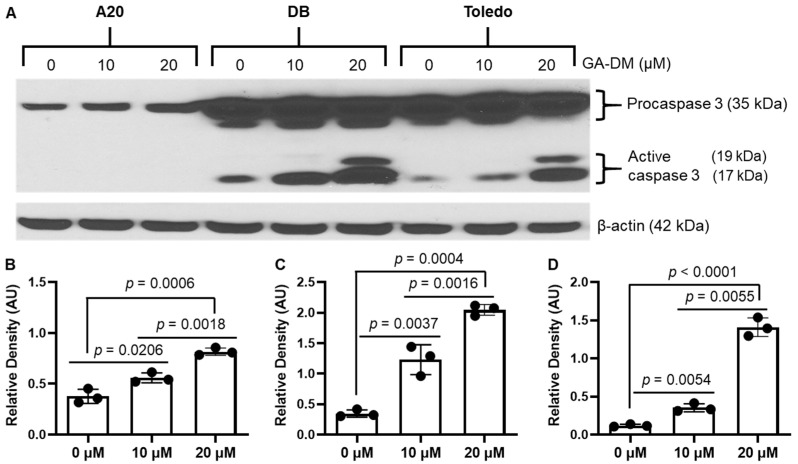
GA-DM treatment increased caspase 3 expression. (**A**) Western blot analysis of whole cell lysates from A20, BD, and Toledo cells. A significant increase in procaspase protein expression in the A20 cell line treated with 10–20 µM of GA-DM. GA-DM treatment induced a dose-dependent increase of active caspases 3 proteins in DB and Toledo cells. β-actin was used as a loading control. (**B**) Quantitative analysis of procaspase 3 in A20 cells by ImageJ software. (**C**,**D**) Quantitative analysis of active caspases (17 kDa) in DB and Toledo cells, respectively.

As mentioned above, GA-DM has been tested in melanoma cells using a known apoptosis inducer, staurosporine, as a positive control [16], suggesting that GA-DM can activate caspases. To examine the mechanism(s) of GA-DM-mediated anti-proliferative activity in human and mouse lymphomas, we first performed a Western blot analysis for caspase 3 (Figure 2A). β-actin was used as a loading control. The results show the presence of cleaved caspase 3 protein in DB and Toledo cells, suggesting that GA-DM induces apoptotic cell death by the activation and processing of caspases into a number of catalytic subunits in human B-cell lymphoma lines (Figure 2). Densitometric analysis by ImageJ showed that GA-DM induced the dose-dependent cleavage of caspases, and active caspase products were detected in DB and Toledo cells (Figure 2B,C), but GA-DM did not affect caspase processing in mouse A20 cells (Figure 2D).

**Figure 3:**
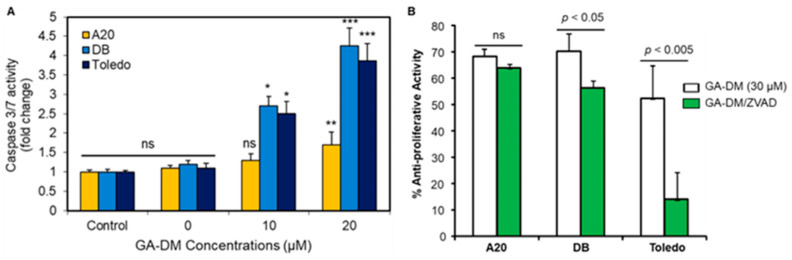
Caspase activities and inhibition by Z-VAD-FMK. (**A**) GA-DM treatment activates caspase 3/7 in B-cell lymphoma lines. Caspase activity was measured in cells treated with GA-DM (20 μM) or vehicle alone for 24 h at 37 °C in 96-well plates as described. Each plate was equilibrated to 22 °C, followed by the addition of Caspase-Glo^®^ 3/7 reagents as per the manufacturer’s recommendations. Luminescence was recorded 30 min after adding the reagent. “Control” indicates the normalized RLU. The figure shown is representative of at least three independent experiments with similar patterns, and error bars represent ± standard deviations of the average. ns = no significant difference; * *p* < 0.001 vs. control; ** *p* < 0.05 vs. control; *** *p* < 0.0001 vs. control. (**B**) Cells were also treated with 30 µM of GA-DM in the presence or absence of Z-VAD-FMK (50 µM) for 24 h and subjected to an MTS cell viability assay. Data suggests that caspase inhibition significantly reversed cell death in both the DB and Toledo cells while having no significant difference in the A20 cells. ns = no significant difference.

As caspases are the major mediators of apoptosis, we assessed the activity of caspases 3 and 7 in treated and untreated mouse and human lymphoma cells using Promega Caspase-Glo^®^ 3/7 reagents as described. A significant increase in the caspase catalytic activities of caspase 3/7 were found in DB and Toledo cells (3–4-fold) after GA-DM treatment (Figure 3A). By contrast, caspase 3/7 activities were not significantly altered in A20 lymphoma cells treated with 10 μM of GADM. However, 20 μM of GA-DM treatment increased caspase 3/7 activities in a statistically significant manner.

A20, DB, and Toledo cells were also treated with GA-DM (30 μM) in the presence or absence of the caspase inhibitor Z-VAD-FMK (50 μM) for 24 h and subjected to an MTS cell viability assay. GA-DM-mediated anti-proliferative activity was significantly reduced in the presence of Z-VAD-FMK in the DB and Toledo human lymphoma cells (Figure 3B). No significant differences in anti-proliferative activities in A20 cells were noted when Z-VAD-FMK was added in the test. These data suggest that GA-DM may differentially induce caspase-mediated cell death in human and mouse B-cell lymphomas. These data also indicate that caspase inhibition significantly reversed cell death in both the DB and Toledo cells while having no significant effect in A20 mouse lymphoma cells.

### 3.3. GA-DM Induces Autophagy in Mouse A20 Lymphoma Cells

**Figure 4:**
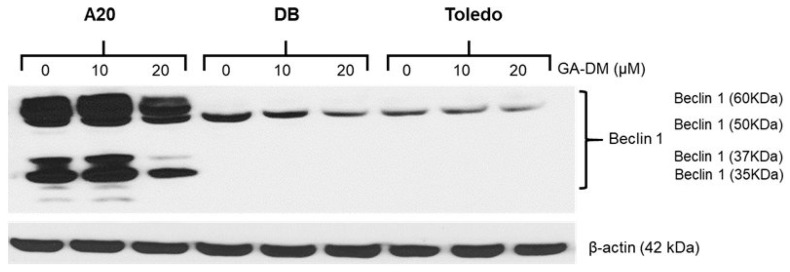
Western blot analysis of whole-cell lysates for Beclin-1 from A20, DB, and Toledo cells. The results show the increased expression of multiple forms of Beclin-1 in A20 cells. There is a slight increase in Beclin-1 protein expression in the A20 cell line at the 10 µM concentration and a marked reduction in Beclin-1 expression at the 20 µM concentration. The molecular weight of Beclin-1 is around 60 kDa, but it can appear as a range of values depending on the antibody and the method used to detect it. Beclin-1 can also be cleaved into fragments of 35, 37, and 50 kDa during apoptosis and cell death. The observed molecular weight of the protein may also vary from the listed predicted molecular weight due to post-translational modifications, post-translational cleavages, relative charges, and other experimental factors. The reduction of Beclin-1 in A20, DB and Toledo cells following the 20 µM GA-DM treatment suggests excessive autophagy and cell death in these B-cell lymphoma lines. GA-DM had minimal effect in altering autophagic proteins in human B-cell lymphoma cells, whereas higher levels of Beclin-1 following the 10 µM GA-DM treatment were found in mouse A20 lymphoma cells.

Western blot analysis of A20 cell lysates showed a significant increase in Beclin-1 protein expression in the A20 cell line at the 10 µM concentration and slightly reduced expression of Beclin-1 at the 20 µM concentration (Figure 4). The reduction in Beclin-1 can be attributed to the possible binding of Beclin-1 to Bcl-2 during A20 cell death at the 20 µM concentration. Interestingly, 10 µM of GA-DM had no statistically significant effect in altering autophagic proteins in the DB and Toledo human B-cell lymphoma cells. The data also showed no significant increase in Beclin-1 protein expression in the A20 cell line at the 20 µM concentration, suggesting that human and mouse cells reacted differently to the GA-DM triterpenoid.

### 3.4. Effects of GA-DM on the Mitochondrial Membrane Potential in Mouse and Human Lymphoma Cells

**Figure 5:**
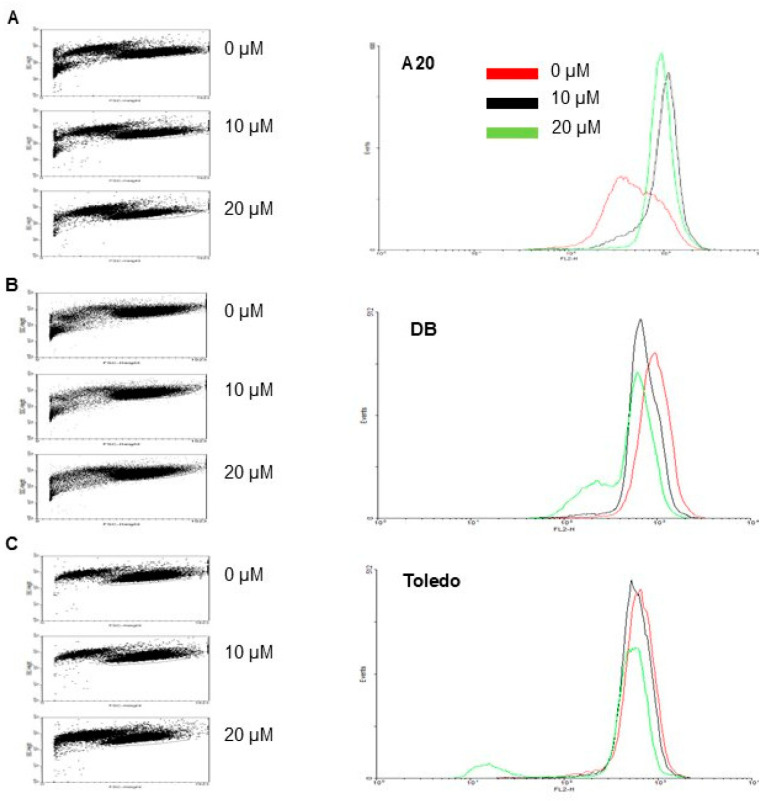
TMRE mitochondrial integrity assay. Cells (A20, DB, and Toledo) were treated with 0, 10, and 20 µM GA-DM overnight and co-incubated with TMRE for 30 min. TMRE penetrates intact polarized mitochondrial membranes and can be detected through flow cytometry. (**A**) GA-DM treatment does not alter mitochondrial membrane integrity in the mouse A20 cell line. (**B**,**C**) GA-DM treatment disrupts the mitochondrial membrane potential in the DB and Toledo cell lines, with the greatest effect on Toledo cells at the 20 µM concentration.

Mitochondrial integrity assays were performed using TMRE. A20, DB, and Toledo cells were treated with 0, 10, and 20 µM GA-DM overnight and co-incubated with TMRE for 30 min. TMRE penetrates intact polarized mitochondrial membranes and can be detected through flow cytometry. TMRE staining indicates that GA-DM treatment does not alter mitochondrial membrane integrity in the mouse A20 cell line (Figure 5). However, GA-DM treatment disrupts the mitochondrial membrane potential in the DB and Toledo cell lines, with the greatest effect on Toledo at the 20 µM concentration. These data suggest that GA-DM differentially affects the mitochondrial potential in mouse and human lymphoma cells, inducing sensitivity to caspase-mediated cell death in the DB and Toledo cell lines, but not in A20 cells.

### 3.5. In Vivo Antitumor Efficacy of GA-DM

**Figure 6:**
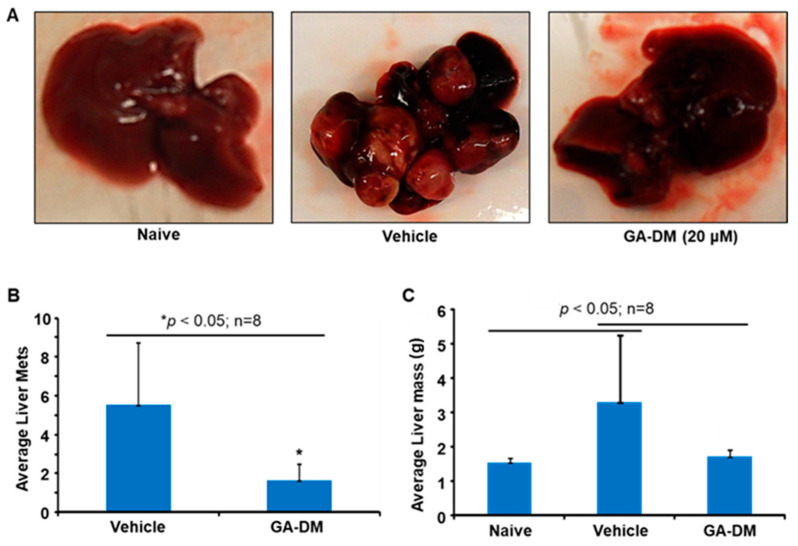
In vivo antitumor efficacy of GA-DM. A20 lymphomas were induced in a BALB/c mouse model as described in the methods. Mice were injected i.p. with GA-DM (50 mg/kg) or control vehicle (DMSO) at days 10 and 21 following the i.v. inoculation of A20 tumor cells as described. (**A**) A20 lymphoma growth and metastasis in BALB/c mice. (**B**,**C**) The average number of liver metastases (Liver Mets) and the total liver mass (in g) were determined upon euthanasia at day 35 post-injection. Results C show that total liver metastases (5.5 ± 3.2 control vs. 1.6 ± 0.87 GA-DM) and gross liver weight (3.2 g ± 1.9 control vs. 1.70 g ± 0.2 GA-DM) were significantly reduced following GA-DM treatment.

A20 lymphoma tumors were induced in a BALB/c mouse model as described in the methods. Mice were injected i.p. with GA-DM (50 mg/kg) or control vehicle (DMSO) at days 10 and 21 following the intravenous (i.v.) inoculation of A20 tumor cells as described. Tumors were collected from naïve, vehicle, and GA-DM treated mice, and photographs were taken (Figure 6A). The average number of liver metastases (Figure 6B) and the total liver mass in grams (Figure 6C) were determined upon euthanasia at day 35 post-injection. The results in Figure 4 show that the total number of liver metastases (5.5 ± 3.2 control vs. 1.6 ± 0.87 GA-DM) and gross liver weight (3.2 g ± 1.9 control vs. 1.70 g ± 0.2 GA-DM) were significantly reduced following GA-DM treatment.

## 4. Discussion

Autophagy and apoptosis constitute functionally distinct mechanisms for the induction of cell survival and death [43,44,45]. While autophagy extends the threshold of stress required for the induction of cell death, it can also remove damaged organelles and support cell survival signals [46,47]. By contrast, apoptosis coincides with biochemical changes such as the activation of effector caspases, including caspase 3, and the breakdown of mitochondrial integrity [45,48]. This apoptosis-associated caspase activation may also lead to the cleavage of various essential pro-autophagic proteins, regulating cell death. Thus, crosstalk between autophagy and apoptosis may occur within the same cell, which determines cell fate and pathophysiological processes, including malignancies. This study has shown that a natural extract of a medicinal mushroom, GA-DM, exhibits anti-proliferative activity against human and mouse B-cell lymphomas. Interestingly, GA-DM activated caspase processing (caspase 3) and death pathways in the human DB and Toledo B-cell lymphomas, but no significant differences were observed in caspase 3 expression in the mouse A20 B-cell lymphoma line. It was further confirmed by using the pan-caspase inhibitor Z-VAD-FMK, which is known to bind to the catalytic site of caspases, as it almost completely reversed the anti-proliferative activity observed in the human DB and Toledo B-cell lymphomas. Recent studies show that evidence is emerging regarding important cell-death-independent, non-apoptotic functions of caspases in metabolic homeostasis that may be of therapeutic value [15,49,50].

Our study suggests that GA-DM induces autophagy and apoptosis in B-cell lymphomas. Specifically, GA-DM activated autophagy in mouse A20 lymphoma cells while showing significant anti-proliferative activity. This suggests that GA-DM may have reduced cell viability by autophagic cell death since no active caspases were detected. However, different scenarios were observed in the human DB and Toledo B-cell lymphoma lines. DB and Toledo cells treated with 10–20 µM concentrations of GA-DM did not have any significant effect on the autophagic protein Beclin-1, but only the 10 µM concentration of GA-DM upregulated active caspase 3. Thus, the autophagic and apoptotic processes induced by GA-DM may have a significant impact in mitigating lymphoma growth. Interestingly, GA-DM had minimal effect in altering autophagic proteins in the DB and Toledo human B-cell lymphoma cells. Autophagy is an important process that consists of the selective degradation of cellular organelles or components and has a close connection to human illnesses like cancer, heart disease, and neurological diseases [51,52,53]. Our data on GA-DM-mediated anti-proliferative activity suggests that autophagy may have highly context-specific functions in regulating cell death as well as many other aspects of physiological functions in vivo. The anti-proliferative activity of GA-DM and the detection of Beclin-1 in A20 cells suggest characteristic features of autophagy, not apoptosis. Autophagy-dependent cell death may also be important in compensating when apoptosis is impaired, as observed in A20 cells, which showed a reduction in cell viability in the absence of active caspase 3. During the process of selective autophagy, damaged and/or redundant organelles like mitochondria, lysosomes, nuclei, and proteasomes are selectively recycled [53]. However, autophagy could be nonselective, where bulky portions of the cytoplasm are degraded upon stress and play important roles in regulating cellular fate in health and disease [53,54]. The crosstalk between autophagy and various forms of cell death makes it difficult to distinguish the specific roles of autophagy in mediating cell survival and death. Apoptosis resistance in developing novel cancer therapies has been a tremendous challenge with current chemotherapy. Thus, the induction of an interplay between autophagy and apoptosis by GA-DM could be exploited.

The autophagy machinery required to mediate cell death may differ from that promoting cell survival. Beclin-1 is expressed in many human and murine tissues and is localized primarily within cytoplasmic structures, including the ER, mitochondria, and the perinuclear membrane. Studies suggest that the induction of autophagy may demonstrate tumor suppressive qualities, whereas a deficiency in autophagy may lead to cellular transformation and tumor development. However, in some cases, malignant cells can also exploit the benefits of autophagy by inducing cell survival associated with a stressful tumor microenvironment as well as toxicity caused by chemotherapeutics. Resistance of cancer cells to treatment can be associated with both autophagy and inhibition of the more common apoptosis cell-death pathway [55,56]. Apoptosis resistance in developing novel cancer therapies has been a tremendous challenge with current chemotherapies, thus the induction of an interplay between autophagy and apoptosis by GA-DM could be exploited. GA-DM treatment differentially influenced the mitochondrial membrane potential in mouse and human lymphoma cells. The TMRE staining suggested that GA-DM did not significantly affect mitochondrial membrane integrity in the mouse A20 lymphoma cell line, supporting our findings that a caspase-activation breakdown of the mitochondrial potential was absent. While autophagy plays a protective role in cells, disruption of autophagy mechanisms or excessive autophagic flux may lead to cell death [57,58,59]. Despite recent progress in the study of the regulation and underlying molecular mechanisms of autophagy, multiple questions remain unanswered regarding autophagic cell death in malignancies. The mitochondrial integrity assay using TMRE showed that GA-DM treatment disrupted the mitochondrial membrane potential in the DB and Toledo cell lines in a dose-dependent manner. This study suggests that the triterpenoid GA-DM differentially influences mitochondrial membrane potential in mouse and human lymphoma cells, possibly by regulating Beclin-1 expression in A20 cells and caspase 3 in DB and Toledo cells. Mitochondria could also be thought of as the victims of autophagy, as well as important regulators of signaling pathways that eventually cause autophagy, as suggested by others. Thus, mitochondria could contribute to survival functions of autophagy. Additionally, activation of autophagy could lead to the removal of damaged organelles or components of the issue in vivo and interfere with apoptosis, protecting against tissue damage. Understanding the regulation of selective or non-selective autophagy in malignant diseases will provide deeper insights into the pathway and open up potential therapeutic avenues.

GA-DM treatment reduced the overall tumor burden in a mouse model of B-cell lymphoma. Analysis of the tumors showed reduced liver metastasis and enhanced recovery in mice by GA-DM treatment. In addition to maintaining intracellular energy and metabolic homeostasis, autophagy can mediate cell death under certain conditions. The expression level of Beclin-1 is thought to be a key determinant as to whether cells are resistant to apoptosis or autophagy during tumorigenesis and chemotherapy. Thus, the reduced expression of Beclin-1 could decrease the autophagic removal of damaged organelles, facilitating cell death and tumor clearance. While an elevation in Beclin-1 expression inhibits tumorigenesis, excessive autophagy promotes apoptosis [60]. It is also known that suppression of apoptosis induces autophagy, while autophagy inhibition causes apoptosis [61,62]. The molecular mechanisms that lead to the demise of the cell during autophagy-dependent cell death are still poorly understood. As mentioned earlier, autophagy can function as a cell survival or a cell death mechanism. Data from Western blot analyses suggests that the expression level of Beclin-1 is reduced at higher concentrations of GA-DM in A20 cells. Since Beclin-1 is known to be a Bcl-2-interacting partner, binding of Beclin-1 to the survival protein Bcl-2 at higher concentration of GA-DM may have reduced Beclin-1 expression in A20 cells. Detection of increased caspase 3/7 activities in A20 cells at higher concentration of GA-DM (20 μM) also support this hypothesis of autophagy-mediated cell death. Identifying the regulatory mechanisms involved in the crosstalk between apoptosis and autophagy and tumor cell fate is essential for creating optimal chemotherapeutics and inhibiting cancer cell growth. Combination approaches utilizing GA-DM with chemotherapy in patients not able to receive more aggressive second-line treatment regimens should be explored for further clinical translation.

## 5. Possible Clinical Implications and Limitations of GA-DM Treatment

Targeting autophagy in malignancies may provide new opportunities for drug development; however, more potent and specific inhibitors of autophagy are needed. While the role of autophagy and its regulation in malignant cells continues to emerge, the current study suggests that new reagents need to be tested to refine strategies to modulate autophagy for therapeutic advantages. Thus, autophagy drugs may be used to treat a variety of diseases, including cancer, neurodegenerative disorders, and metabolic diseases. While autophagy can be both a tumor suppressor and a tumor promoter, inhibiting autophagy may help eliminate tumor cells that are resistant to chemotherapy. However, autophagy can also affect normal cells, making them more sensitive to chemotherapy. Our study suggests that excessive autophagy may induce apoptosis, clearing tumor cells in the microenvironment. There are also limitations in autophagy drugs, which include their lack of specificity, potential for off-target effects, difficulty in accurately measuring autophagy modulation in vivo, and the possibility of inducing resistance in cancer cells, depending on the stage of the disease and the specific autophagy target being manipulated. Autophagy may also interact with other anti-cancer therapies. Although there are limitations in existing therapeutic approaches against malignant diseases, new drugs could be developed that target autophagy and that can help prevent or cure diseases by degrading dysfunctional proteins or pathogens.

## Data Availability

The data used to support the findings of this manuscript are available from the corresponding authors upon reasonable written request after publication.
